# Risk Factors of Severe *Clostridioides difficile* Infection; Sequential Organ Failure Assessment Score, Antibiotics, and Ribotypes

**DOI:** 10.3389/fmicb.2022.900681

**Published:** 2022-05-12

**Authors:** Min Hyuk Choi, Dokyun Kim, Seok Hoon Jeong, Hyuk Min Lee, Heejung Kim

**Affiliations:** ^1^Department of Laboratory Medicine and Research Institute of Bacterial Resistance, Yonsei University College of Medicine, Gangnam Severance Hospital, Seoul, South Korea; ^2^Department of Laboratory Medicine and Research Institute of Bacterial Resistance, Yonsei University College of Medicine, Severance Hospital, Seoul, South Korea; ^3^Department of Laboratory Medicine and Research Institute of Bacterial Resistance, Yonsei University College of Medicine, Yongin Severance Hospital, Yongin, South Korea

**Keywords:** *Clostridioides difficile*, CDI, sequential organ failure assessment score, ribotyping, antibiotics, machine learning

## Abstract

We aimed to determine whether the Sequential Organ Failure Assessment (SOFA) score predicts the prognosis of patients with *Clostridioides difficile* infection (CDI). In addition, the association between the type of antibiotic used and PCR ribotypes was analyzed. We conducted a propensity score (PS)-matched study and machine learning analysis using clinical data from all adult patients with confirmed CDI in three South Korean hospitals. A total of 5,337 adult patients with CDI were included in this study, and 828 (15.5%) were classified as having severe CDI. The top variables selected by the machine learning models were maximum body temperature, platelet count, eosinophil count, oxygen saturation, Glasgow Coma Scale, serum albumin, and respiratory rate. After propensity score-matching, the SOFA score, white blood cell (WBC) count, serum albumin level, and ventilator use were significantly associated with severe CDI (*P* < 0.001 for all). The log-rank test of SOFA score ≥ 4 significantly differentiated severe CDI patients from the non-severe group. The use of fluoroquinolone was more related to CDI patients with ribotype 018 strains than to ribotype 014/020 (*P* < 0.001). Even after controlling for other variables using propensity score matching analysis, we found that the SOFA score was a clinical predictor of severe CDI. We also demonstrated that the use of fluoroquinolones in hospital settings could be associated with the PCR ribotype in patients with CDI.

## Introduction

*Clostridioides difficile* is a toxin-producing and spore-forming Gram-positive anaerobic bacterium that may colonize and cause infections in the human intestinal tract due to dysbiosis resulting from antibiotic treatment (McDonald et al., 2018). *C. difficile* infection (CDI) is a leading cause of healthcare-associated diarrhea and is a global concern that can exacerbate patient conditions and increase morbidity and mortality (Davies et al., 2014; Lessa et al., 2015; McDonald et al., 2018). Since CDI is associated with worse clinical outcomes in patients, several indicators, including laboratory results like WBC count and serum albumin level, patient symptoms, and hemodynamic changes have been proposed to discriminate disease severity (Surawicz et al., 2013; McDonald et al., 2018; Johnson et al., 2021). Clinical guidelines recommend treatment regimens for patients with CDI based on disease severity classified by these indicators (Johnson et al., 2021; van Prehn et al., 2021). For clinicians, early prediction of patients with CDI at risk of severe disease is important for decision-making and disease management. However, there are no standardized and validated predictive indicators for identifying high-risk groups prior to disease progression.

The Sequential Organ Failure Assessment (SOFA) score is an objective, early obtainable value that is widely used to assess and/or predict a patient’s prognosis in infectious disease research. SOFA is used as a measure of sepsis-related organ dysfunction, which can be identified as an acute change of two or more points in the total score (Singer et al., 2016) and is also useful in predicting the prognosis of critically ill patients (Vincent et al., 1998). However, validation studies applying the SOFA score to grade the severity of CDI in patients are still lacking.

The aim of this study was to determine whether the SOFA score predicts the prognosis of patients with CDI at the time of diagnosis. We then identified the top variables, including components of the SOFA score with the largest impact on the prediction of severe CDI *via* machine learning (ML) analysis. ML techniques were used to evaluate the importance of clinical indicators along with conventional statistical approaches (Taylor et al., 2016; Chiew et al., 2020; Roimi et al., 2020). Furthermore, we also analyzed polymerase chain reaction (PCR) ribotyping of clinical *C. difficile* isolates to evaluate their association with the type of antibiotic used in the patient.

## Patients and Methods

### Study Population and Data Collection

We retrospectively extracted data from all adult patients (≥18 years of age) with confirmed CDI from January 2011 to June 2021 using the Severance Clinical Research Analysis Portal. This electronic health records data collection program with information from two university tertiary hospitals and one secondary hospital in South Korea has been in existence since 2006. CDI was defined as having three or more loose stools over a 24-h period and positive for *C. difficile* toxin on a nucleic acid amplification test (for the toxin B gene), or rapid antigen/toxin enzyme immunoassay. During the study period, 12,290 patients were confirmed as having CDI. Multiple positive results of CDI in the same patients, except for the first test record, were excluded (*n* = 5,782). After selecting the first confirmed cases from each of the 6,508 patients, we excluded 1,171 patients who had no demographic information.

We collected patient-level data, including demographics, underlying comorbidities, date of CDI diagnosis, and date of death. We obtained the most abnormal values within 72 h of a hospital visit and CDI diagnosis, by extracting both the maximum and minimum values of laboratory test results and vital signs. In addition, we investigated the use of mechanical ventilation, vasopressors, and the lowest Glasgow Coma Scale (GCS) within the 72-h period. The microbiological test results and history of antibiotic use within 60 days before CDI diagnosis were also recorded. We performed PCR ribotyping of 1,464 isolates collected from patients who could provide stool samples for *C. difficile* culture, as described in our previous studies (Kim et al., 2011, 2021).

### Definition of *Clostridioides difficile* Infection

According to McDonald et al. (2007), severe CDI (outcome of interest) was defined as the presence of one or more of the following: intensive care unit admission, need for interventional surgery, and death within 30 days of diagnosis (McDonald et al., 2007). Progression-free survival (PFS) refers to the duration of time that CDI patients remain non-severe on treatment.

### Propensity Score-Matched Analysis

To reduce selection bias that affects clinical outcomes depending on the difference in the patient’s baseline condition at the time of hospital visit, we conducted a PS-matched study and conditional logistic regression using MatchIt package (Heinze and Jüni, 2011). We selected six variables including age, sex, the Charlson comorbidity index (Charlson et al., 1987), WBC count, serum albumin, and SOFA score (*P* < 0.001 for all) at the time of hospital visit for adjustment by univariate analysis (Brookhart et al., 2006; Austin, 2008). We then performed a PS-matched analysis by attempting to match each patient with severe CDI to a non-severe CDI (1:2 match) using the nearest-neighbor-matching method. A match occurred when the difference in logits of PS was less than 0.2 times the standard deviation of scores.

### Statistical Analysis

We described the patient’s characteristics using numbers and percentages for categorical variables, medians, and interquartile ranges (IQRs) for continuous variables. The statistical significance between groups was tested with Fisher’s exact test for qualitative data and the Mann–Whitney *U* test for quantitative data. We used conditional logistic regression for univariate and multivariate analysis between groups of patients with severe and non-severe CDI. Dependent variables included in the multivariate analysis were selected based on the statistical significance provided by univariate analysis. We employed the Kaplan-Meier estimator to analyze PFS, and differences between groups were assessed using the log-rank test.

All reported *P*-values were two-tailed, and statistical significance was assumed if *P* < 0.05. Statistical analyses were performed using R statistical software version 4.1 (R Studio, Inc., Boston, MA, United States).

### Machine Learning Analysis

Before modeling, all continuous variables were standardized, and missing values were imputed using the median value (Chiew et al., 2020). The dataset was randomly split at a ratio of 4:1 for the training and test sets. For each ML model, hyperparameter tuning was performed through a grid search and fivefold cross-validation. Candidate models were trained using the K-nearest neighbor (KNN), decision tree, random forest, light gradient boosting machine (LightGBM), eXtreme gradient boosting (XGBoost), support vector machine (SVM), and artificial neural network algorithms (ANN). Each model with the highest area under the receiver operator characteristic curve (AUROC) with 95% confidence intervals (CIs), accuracy, and F1 score (the harmonic mean of precision and recall) was generated (LeDell et al., 2015; Taylor et al., 2016). ML analysis was performed using Python programming software version 3.7.12 (Python Software Foundation, Wilmington, DE, United States).

### Ethics Statement

The Institutional Review Board at Severance Hospital, affiliated with the Yonsei University Health System (3-2021-0508), approved this study.

## Results

### Before Propensity Score Matching

A total of 5,337 adult patients with CDI between January 2010 and June 2021 were included in this study. The demographic and clinical characteristics of patients with severe and non-severe CDI are summarized in Table 1. The median age of the patients was 65 years (IQR, 51–75 years), and 828 (15.5%) had severe CDI. The 1,464 (27.4%) isolates for PCR ribotyping produced 88 distinct *C. difficile* ribotypes. Among them, ribotype 014/020 (R014/020) accounted for the largest proportion (16.3%), followed by R018 (16.0%). Other ribotypes were observed at less than 10.0% each, and hypervirulent strains accounted for only 3.7% of R078 and 0.9% of R027. At baseline, severe and non-severe CDI groups showed statistically significant differences in most variables of severity and epidemiologic characteristics, except for PCR ribotype and body temperature. Patients in the severe CDI group were older, mostly male, were more often included in hospital-onset disease, and had a higher Charlson comorbidity index than those in the non-severe CDI group (*P* < 0.001 for all). Similarly, the severe CDI group had a higher baseline SOFA score, WBC count, serum creatinine level, and lower systolic and diastolic blood pressures and serum albumin than the non-severe CDI group (*P* < 0.001 for all).

**TABLE 1:** Baseline characteristics of patients with CDI, before propensity-score matching.

	Total	Severe CDI	Non-severe CDI	*P*-value
	(*n* = 5,337)	(*n* = 828)	(*n* = 4,509)	
Age, y	65 [51–75]	70 [59–78]	64 [50–75]	<0.001
Sex				<0.001
Female	2,617 (49.0%)	346 (41.8%)	2,271 (50.4%)	
Male	2,720 (51.0%)	482 (58.2%)	2,238 (49.6%)	
Hospital onset disease	4,011 (75.2%)	701 (84.7%)	3,310 (73.4%)	<0.001
**Charlson comorbidity index**	4 [3–6]	5 [4–7]	4 [3–6]	<0.001
Solid organ cancer	1,733 (32.5%)	320 (38.6%)	1,413 (31.3%)	<0.001
Kidney disease	372 (7.0%)	102 (12.3%)	270 (6.0%)	<0.001
Chronic lung disease	137 (2.6%)	35 (4.2%)	102 (2.3%)	<0.001
Diabetes mellitus	796 (14.9%)	161 (19.4%)	635 (14.1%)	<0.001
Proton pump inhibitor use	704 (13.2%)	141 (17.0%)	563 (12.5%)	<0.001
Enteral feeding	121 (2.3%)	40 (4.8%)	81 (1.8%)	<0.001
**Ribotype (available strains)**	1,464 (27.4%)	220 (26.6%)	1,244 (27.6%)	0.480
R001	56 (3.8%)	11 (5.0%)	45 (3.6%)	
R002	124 (8.5%)	19 (8.6%)	105 (8.4%)	
R012	75 (5.1%)	8 (3.6%)	67 (5.4%)	
R014/020	238 (16.3%)	26 (11.8%)	212 (17.0%)	
R017	95 (6.5%)	17 (7.7%)	78 (6.3%)	
R018	234 (16.0%)	39 (17.7%)	195 (15.7%)	
R046	102 (7.0%)	17 (7.7%)	85 (6.8%)	
Other types	540 (36.9%)	83 (37.7%)	457 (36.7%)	
**Variables within 72 h at the time of admission**				
SOFA score at hospital visit	2 [0–5]	5 [2–8]	2 [0–4]	<0.001
Minimum systolic blood pressure (mmHg)	101 [98–111]	99 [98–110]	101 [98–111]	<0.001
Minimum diastolic blood pressure (mmHg)	60 [52–74]	60 [48–78]	61 [52–73]	<0.001
Maximum body temperature (^°^C)	37.5 [37.1–38.3]	37.5 [37.0–38.3]	37.5 [37.1–38.3]	0.435
Minimum serum albumin (g/dL)	2.9 [2.5–3.3]	2.5 [2.2–2.8]	2.9 [2.5–3.4]	<0.001
Maximum white blood cell count (10^9^/L)	9.7 [6.3–12.7]	10.6 [7.9–16.4]	9.3 [6.2–12.3]	<0.001
Maximum serum creatinine (mg/ml)	0.8 [0.6–1.4]	1.1 [0.7–2.2]	0.8 [0.6–1.2]	<0.001
**Variables within 72 h at the time of CDI diagnosis**				
SOFA score at CDI diagnosis	2 [0–4]	5 [2–8]	1 [0–4]	<0.001
Increased in SOFA score ≥ 2 points consequent to CDI	546 (10.2%)	157 (19.0%)	389 (8.6%)	<0.001
Minimum systolic blood pressure (mmHg)	100 [98–108]	99 [98–104]	100 [99–109]	<0.001
Minimum diastolic blood pressure (mmHg)	60 [51–73.5]	58 [45–92]	60 [52–72]	0.001
Maximum body temperature (^°^C)	37.7 [37.2–38.5]	37.8 [37.2–38.5]	37.7 [37.2–38.5]	0.618
Minimum serum albumin (g/dL)	3.0 [2.5–3.5]	2.6 [2.2–3.0]	3.0 [2.6–3.5]	<0.001
Maximum white blood cell count (10^9^/L)	9.7 [6.4–12.6]	10.5 [7.6–14.9]	9.4 [6.2–12.3]	<0.001
Minimum eosinophil count (10^9^/L)	0.0 [0.0–0.1]	0.0 [0.0–0.1]	0.1 [0.0–0.1]	<0.001
Maximum C-reactive protein (mg/L)	56.5 [14.7–125.0]	91.7 [38.9–164.8]	49.0 [12.1–118.1]	<0.001
Maximum total bilirubin (mg/dL)	0.6 [0.4–1.0]	0.7 [0.5–1.3]	0.6 [0.4–0.9]	<0.001
Minimum platelet count (10^9^/L)	190.0 [117.0–271.0]	149.0 [93.0–235.0]	196.0 [125.0–276.0]	<0.001
Maximum serum creatinine (mg/ml)	0.8 [0.6–1.4]	1.1 [0.7–2.2]	0.8 [0.6–1.3]	<0.001

*CDI, Clostridioides difficile infection; SOFA, sequential organ failure assessment.*

*Data are presented as number (%) or medians [interquartile range (IQR)].*

### After Propensity Score Matching

After PS-matching, baseline characteristics including age, sex, SOFA score, minimum serum albumin, and maximum WBC count of both groups were well-balanced in 767 pairs at a 1:2 ratio and were not statistically different (Table 2). However, both the SOFA score at the time of CDI diagnosis and the increased rate of the SOFA score by 2 or more points were significantly higher in the severe CDI group than in the non-severe CDI group (*P* < 0.001, both). In addition, minimum systolic blood pressure, minimum serum albumin, maximum WBC count, minimum eosinophil count, maximum C-reactive protein (CRP), and maximum total bilirubin still differed significantly.

**TABLE 2:** Baseline characteristics of patients with CDI, after propensity-score matching.

	Severe CDI	Non-severe CDI	*P-*value
	(*n* = 767)	(*n* = 1,534)	
Age, y	70 [59–77]	70 [59–77]	0.586
Sex			0.800
Female	326 (42.5%)	642 (41.9%)	
Male	441 (57.5%)	892 (58.1%)	
Hospital onset disease	650 (84.7%)	1,247 (81.3%)	0.066
**Charlson comorbidity index**	5 [4–7]	5 [4–7]	0.772
Solid organ cancer	309 (40.3%)	578 (37.7%)	0.244
Kidney disease	87 (11.3%)	168 (11.0%)	0.833
Chronic lung disease	32 (4.2%)	47 (3.1%)	0.210
Diabetes mellitus	149 (19.4%)	319 (20.8%)	0.475
Proton pump inhibitor use	126 (16.4%)	221 (14.4%)	0.224
Enteral feeding	31 (4.0%)	47 (3.1%)	0.271
**Ribotype (available strains)**	203 (26.5%)	425 (27.7%)	
R001	11 (5.4%)	19 (4.5%)	0.846
R002	16 (7.9%)	39 (9.2%)	
R012	8 (3.9%)	17 (4.0%)	
R014/020	26 (12.8%)	66 (15.5%)	
R017	16 (7.9%)	55 (12.9%)	
R018	36 (17.7%)	79 (18.6%)	
R046	16 (7.9%)	23 (5.4%)	
Other types	74 (36.5%)	127 (29.9%)	
**Variables within 72 h at the time of hospital visit**			
SOFA score at hospital visit	3 [2–7]	3 [2–7]	0.164
Minimum systolic blood pressure (mmHg)	100 [98–110]	100 [98–109]	0.517
Minimum diastolic blood pressure (mmHg)	60 [49–77]	58 [49–75]	0.349
Maximum body temperature (^°^C)	37.7 [37.4–38.2]	37.8 [37.2–38.5]	0.090
Minimum serum albumin (g/dL)	2.6 [2.4–2.9]	2.7 [2.4–3.1]	0.091
Maximum white blood cell count (10^9^/L)	10.5 [7.5–15.4]	10.3 [7.2–13.7]	0.095
Maximum serum creatinine (mg/ml)	1.0 [0.7–2.0]	1.0 [0.6–1.8]	0.198
**Variables within 72 h at the time of CDI diagnosis**			
SOFA score at CDI diagnosis	4 [2–8]	3 [1–6]	<0.001
Increased in SOFA score ≥ 2 points consequent to CDI	153 (19.9%)	137 (8.9%)	<0.001
Minimum systolic blood pressure (mmHg)	99 [98–105]	100 [98–108]	<0.001
Minimum diastolic blood pressure (mmHg)	58 [46–92]	58 [50–71]	0.609
Maximum body temperature (^°^C)	37.8 [37.2–38.4]	37.8 [37.3–38.5]	0.050
Minimum serum albumin (g/dL)	2.6 [2.3–3.1]	2.7 [2.4–3.2]	<0.001
Maximum white blood cell count (10^9^/L)	10.5 [7.4–14.1]	10.2 [6.9–13.6]	0.019
Minimum eosinophil count (10^9^/L)	0.0 [0.0–0.1]	0.0 [0.0–0.1]	0.014
Maximum C-reactive protein (mg/L)	89.6 [37.1–161.9]	77.0 [23.2–148.4]	<0.001
Maximum total bilirubin (mg/dL)	0.7 [0.5–1.3]	0.7 [0.4–1.1]	<0.001
Minimum platelet count (10^9^/L)	156.0 [94.0–241.0]	161.0 [98.0–251.0]	0.051
Maximum serum creatinine (mg/ml)	1.1 [0.7–2.0]	1.0 [0.7–1.9]	0.361

*CDI, Clostridioides difficile infection; SOFA, sequential organ failure assessment.*

*Data are presented as number (%) or medians [interquartile range (IQR)].*

We used univariate and multivariate analysis with conditional logistic regression to identify risk factors for severe CDI (Table 3). After PS matching, seven independent variables were significant indicators of severe CDI in the univariate analysis. Since the SOFA score at the time of CDI diagnosis and the increase in SOFA score by more than 2 points had multicollinearity, it was analyzed separately by the different models in multivariate analysis. In multivariate analysis model 1, the SOFA score (adjusted odds ratio [aOR], 1.16; 95% CI, 1.11–1.20; *P* < 0.001), maximum WBC count (aOR, 1.01; 95% CI, 1.00–1.02; *P* < 0.001), minimum serum albumin (aOR, 0.65; 95% CI, 0.52–0.51; *P* < 0.001), and ventilator use (aOR, 5.49; 95% CI, 2.23–13.55; *P* < 0.001) were associated with severe CDI. In multivariate analysis model 2, increases of more than 2 points in SOFA scores were also found to be significantly associated with severe CDI, even after adjusting for other variables (aOR, 2.29; 95% CI, 1.68–3.11; *P* < 0.001). The ribotype of the strains was not associated with severe CDI.

**TABLE 3:** Univariate and multivariate analysis using conditional logistic regression of risk factors of severe CDI, after propensity-score matching.

	Univariate analysis		Multivariable analysis model 1		Multivariable analysis model 2	
			
	OR (95% CI)	*P-*value	OR (95% CI)	*P-*value	OR (95% CI)	*P-*value
Age, y	1.00 (0.99–1.01)	0.795				
Male sex	0.97 (0.82–1.16)	0.763				
Hospital onset disease	1.29 (1.01–1.63)	0.038	1.26 (0.95–1.66)	0.109	1.14 (0.86–1.51)	0.361
Charlson comorbidity index	1.00 (0.96–1.05)	0.900				
Solid organ cancer	1.13 (0.94–1.35)	0.207				
Kidney disease	1.04 (0.79–1.38)	0.773				
Chronic lung disease	1.38 (0.87–2.18)	0.170				
Diabetes mellitus	0.92 (0.73–1.14)	0.435				
Proton pump inhibitor use	1.17 (0.92–1.48)	0.204				
Enteral feeding	1.34 (0.84–2.14)	0.219				
**Ribotype**						
Other types	Reference					
R001	1.32 (0.34–5.04)	0.689				
R002	0.45 (0.16–1.29)	0.138				
R012	0.87 (0.22–3.47)	0.847				
R014/020	0.89 (0.37–2.14)	0.792				
R017	0.60 (0.20–1.81)	0.370				
R018	0.67 (0.30–1.49)	0.325				
R046	1.07 (0.30–3.78)	0.914				
**Variables within 72 h at the time of CDI diagnosis**						
SOFA score	1.18 (1.15–1.22)	<0.001	1.16 (1.11–1.20)	<**0.001**		
Increased in SOFA score ≥ 2 points consequent to CDI	2.51 (1.95–3.23)	< 0.001			2.29 (1.68–3.11)	<** 0.001**
Maximum body temperature (^°^C)	0.94 (0.88–1.01)	0.093				
Maximum white blood cell count (10^9^/L)	0.74 (0.63–0.88)	<0.001	1.01 (1.00–1.02)	<**0.001**	1.01 (1.00–1.02)	**0.026**
Minimum serum albumin (g/dL)	1.01 (1.00–1.02)	<0.001	0.65 (0.52–0.81)	<**0.001**	0.62 (0.50–0.77)	<**0.001**
Minimum eosinophil count (10^9^/L)	0.86 (0.71–1.06)	0.156				
Maximum C-reactive protein (mg/L)	1.00 (1.00–1.01)	0.027	1.00 (0.99–1.00)	0.978	1.00 (0.99–1.00)	0.859
Maximum serum creatinine (mg/ml)	1.00 (0.96–1.05)	0.858				
Ventilator use	13.01 (5.5–3.71)	<0.001	5.49 (2.23–13.55)	<**0.001**	8.42 (3.48–20.37)	<**0.001**

*CDI, Clostridioides difficile infection; OR, odds ratio; CI, confidence interval; SOFA, sequential organ failure assessment.*

*Significant (P < 0.05) variables in the multivariable analysis are indicated in bold.*

### Sequential Organ Failure Assessment Scores in *Clostridioides difficile* Infection Patients and Comparison of the Predictive Models

**FIGURE 1:**
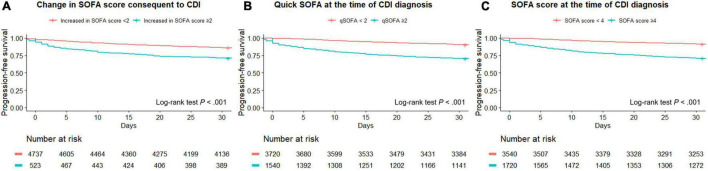
Progression-free survival curves for three types of SOFA SCORES, including changes in SOFA score **(A)**, quick SOFA **(B)**, and SOFA **(C)**.

The optimal cut-off value of the SOFA score for discriminating severe CDI was 4 points, as shown in the AUROC curve (Supplementary Figure 1). Among all patients, the log-rank test of SOFA score ≥ 4 was significantly different in patients with severe CDI from the non-severe group (*P* < 0.001). PFS curves for dichotomized SOFA scores of the two groups are shown in Figure 1. The SOFA, quick SOFA (qSOFA), and change in SOFA score consequent to CDI were significantly different in both groups (*P* < 0.001 for all three indicators).

**FIGURE 2:**
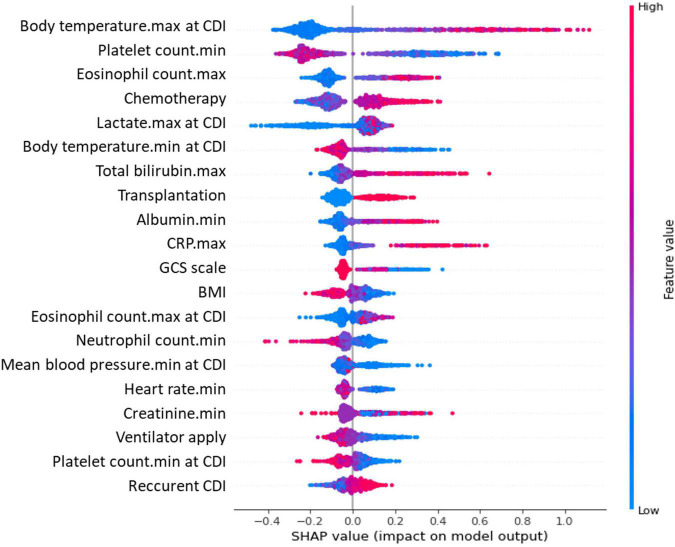
Critical variables with SHAP values for predicting severe CDI. SHAP, Shapley additive explanation; CDI, *Clostridioides difficile* infection; SOFA, sequential organ failure assessment; CRP, C-reactive protein; GCS, Glasgow Coma Scale; BMI, body mass index. A SHAP value summary dot plot of the light gradient boosting model. The color of the SHAP dot represents the value of the feature, and the location of the dot on the X-axis represents the SHAP value. Red dots indicate higher values or affirmative responses, and blue dots indicate the opposite. A positive SHAP value indicates that the variables increase the likelihood of severe CDI.

The predictive performance of the SOFA, qSOFA score, and ML models is summarized in Supplementary Table 1. In the analysis for early discrimination of severe CDI, the SOFA score and the change in SOFA score consequent to CDI showed similar performance (AUROC, 0.732; 95% CI, 0.712–0.751 for both; F1 score, 0.400 for SOFA score and 0.403 for changes in SOFA score ≥ 2), and qSOFA showed relatively inferior performance (AUROC 0.685; 95% CI, 0.665–0.705; F1 score, 0.388). Among the performance of the ML algorithm in the internal test set, the XGBoost classifier showed the highest AUROC value of 0.806 (95% CI, 0.776–0.834), and the LightGBM classifier showed the highest accuracy of 0.859. In addition, the top predictors of ML models for severe CDI are presented. The importance plots of the XGBoost (Supplementary Figure 2) and Shapley additive explanation (SHAP) analysis of the LightGBM classifier (Figure 2) showed the most important indicators used in the ML analysis. Oxygen saturation, respiratory rate, blood urea nitrogen, GCS, and serum albumin were the top predictors in the XGBoost model, and body temperature, platelet count, eosinophil count, chemotherapy within 2 weeks, and serum lactate were selected in LightGBM.

### The Relationship Between the Type of Antibiotic Used and the Main Ribotype of *Clostridioides difficile*

Table 4 shows the comparison between the two most common ribotypes (R014/020 and R018) in this study and the type of antibiotic used within 60 days before CDI diagnosis. In the period from 2011 to 2014, *C. difficile* R018 was the most common strain, with 23.6% of all tested isolates; however, the relative incidence of R014/020 increased and became the most common strain. After adjusting for confounding factors, the history of use of fluoroquinolone was more associated with CDI patients with R018 strains than with R014/020 (aOR, 1.96; 95% CI, 1.31–2.93; *P* < 0.001). The annual incidence of fluoroquinolone prescription per 1,000 inpatient days in our hospitals is illustrated in Supplementary Figure 3 and has continued to decline since 2019.

**TABLE 4:** Comparison between the two most common ribotypes and the type of antibiotic used within 60 days before the CDI diagnosis.

	R014/020	R018	Others		Univariate analysis		Multivariable analysis	
						
	(*N* = 238)	(*N* = 234)	(*N* = 992)	*P-*value	OR (95% CI)	*P-*value	OR (95% CI)	*P-*value
**Period of *C. difficile* isolation**				<0.001				
2011–2014	79 (13.9%)	134 (23.6%)	355 (62.5%)		Reference		Reference	
2015–2018	48 (14.7%)	40 (12.2%)	239 (73.1%)		0.49 (0.30–0.81)	0.006	0.65 (0.38–1.12)	0.124
2019–2021.6	111 (19.5%)	60 (10.5%)	398 (70.0%)		0.32 (0.21–0.48)	<0.001	0.40 (0.25–0.62)	<0.001
Age, y	64 [48–75]	66 [54–75]	67 [53–76]	0.079				
Female sex	119 (50.0%)	100 (42.7%)	499 (50.3%)	0.108				
Hospital onset disease	178 (74.8%)	187 (79.9%)	747 (75.3%)	0.299				
Length of hospital stay	16 [8–34]	29 [13–49]	21 [9–43]	<0.001	1.00 (0.99–1.01)	0.124	1.00 (0.99–1.00)	0.867
**Antimicrobial exposure within 60 days**								
1st-generation cephalosporins	15 (6.3%)	27 (11.5%)	69 (7.0%)	0.040	1.94 (1.00–3.75)	0.049		
2nd-generation cephalosporins	28 (11.8%)	23 (9.8%)	91 (9.2%)	0.483				
3rd-generation cephalosporins	70 (29.4%)	79 (33.8%)	322 (32.5%)	0.572				
4th-generation cephalosporins	20 (8.4%)	43 (18.4%)	77 (7.8%)	<0.001	2.45 (1.39–4.32)	<0.001	1.81 (0.97–3.38)	0.064
ß-lactam/ß-lactamase inhibitor combinations	93 (39.1%)	90 (38.5%)	415 (41.8%)	0.537				
Aminoglycosides	9 (3.8%)	31 (13.2%)	75 (7.6%)	<0.001	3.89 (1.81–8.36)	<0.001	1.97 (0.85–4.58)	0.116
**Fluoroquinolones**	79 (33.2%)	129 (55.1%)	352 (35.5%)	<0.001	2.47 (1.70–3.59)	<0.001	1.96 (1.31–2.93)	<0.001
Carbapenems	38 (16.0%)	51 (21.8%)	205 (20.7%)	0.207				
Charlson comorbidity index	4 [3–5]	5 [3–6]	4 [3–6]	<0.001	1.14 (1.05–1.23)	<0.001	1.10 (1.01–1.19)	0.029

*CDI, Clostridioides difficile infection.*

*Data are presented as number (%) or medians [interquartile range (IQR)].*

## Discussion

We found that the SOFA score calculated with variables within 72 h of CDI diagnosis was statistically associated with patient outcome, even after PS matching and adjustment for other variables.

The observational approach of our study may have led to selection bias. There were systemically significant differences in the following initial parameters of patients between the severe and non-severe CDI groups: age, sex, underlying comorbidities, rate of proton pump inhibitor use and enteral feeding, and the most abnormal values of vital signs and laboratory test results. Differences in the baseline characteristics of patients known to be associated with severe CDI (Bliss et al., 1998; Loo et al., 2011; Surawicz et al., 2013; Abou Chakra et al., 2015; Trifan et al., 2017; McDonald et al., 2018) can act as confounding factors for clinical outcomes. The PS-matched study is a method of designing observational studies that mimic the characteristics of randomized controlled trials, allowing for a similar distribution of the observed baseline covariates between severe and non-severe CDI groups. Therefore, we conducted a multivariate analysis using PS-matched data to minimize selection bias (Austin, 2008; Heinze and Jüni, 2011; Wombwell et al., 2021).

Several scoring systems have been developed to predict the severity of CDI, but none of them have been validated (Barbut and Rupnik, 2012; Kassam et al., 2016; Ahmed et al., 2021), and there is still no consensus indicator that can be used to differentiate disease severity (Bauer et al., 2012; Surawicz et al., 2013; Debast et al., 2014; McDonald et al., 2018). The SOFA score is a widely accepted predictive model for patients with infectious diseases. It is a validated score that can be used to predict the prognosis of individual patients and helps to compare the quality of care between hospitals and standardized studies. We included a large number of cases and attempted to control for confounders, thus ensuring that the SOFA score is related to patients with severe CDI. Furthermore, we also presented a dichotomous cutoff of SOFA scores to predict severe CDI using the AUROC and PFS curves in our study.

In addition to the SOFA score, other variables such as WBC count, serum albumin, and ventilator use were also significantly different between patients with severe and non-severe CDI, which is consistent with prior studies (Surawicz et al., 2013; Abou Chakra et al., 2015; McDonald et al., 2018). Certain *C. difficile* ribotypes, such as R027 and R078, have been shown to be more virulent than others in epidemic settings (He et al., 2013; Hensgens et al., 2013), and fluoroquinolone use was closely correlated with the emergence of CDI due to the resistance of the R027 strain to this antibiotic. However, other studies in non-outbreak settings found that this ribotype did not significantly predict severe CDI (Welfare et al., 2011; Walk et al., 2012; Abou Chakra et al., 2015). In our data, there was no statistical association between *C. difficile* ribotype and severe CDI, where the prevalence of R027 and R078 was less than 5% of the available strains.

Dingle et al. (2017) reported that restriction of fluoroquinolone use reduced the incidence of CDI in an England population-based study, mainly driven by the elimination of fluoroquinolone-resistant isolates. Similarly, in our hospital-based data, fluoroquinolone use was associated with the relative incidence of CDI by major PCR ribotypes and was observed more frequently in patients with CDI due to the R018 strain than the R014/020 strain. All R018 strains had *gyrA* mutations and showed resistance to quinolone, whereas R014/020 strains had a *gyrA* mutation in 8.1% of the isolates. This suggests that the use of fluoroquinolone could act as a selective pressure to induce CDI due to antibiotic-resistant ribotype (Loo et al., 2005; Muto et al., 2007; Kallen et al., 2009; He et al., 2013; Abou Chakra et al., 2015) and a decrease in the annual prescription of these antibiotics in our centers may have influenced the change in the relative incidence of *C. difficile* ribotypes.

Traditional multivariate analysis has fundamental limitations in selecting independent variables to be included in the model owing to the effects of multicollinearity and overfitting issues (Baxt, 1994). Comprehensive data analysis though ML can be utilized in conjunction with conventional statistical analysis to evaluate the adequacy of clinical indicators. Therefore, we investigated 135 covariates in the clinical data, but only six variables were included in the final statistical models through univariate analysis. ML-based models have the advantage of correcting non-linear relationships and multicollinearity of variables, which can provide new insights into various fields of clinical medicine (Baxt, 1994; Wiens and Shenoy, 2018). For example, in the SHAP analysis of this study, both the maximum and minimum values of body temperature were selected as the top predictors. The maximum value of body temperature was directly proportional to the risk of severe CDI, while the minimum value showed a negative correlation, which is difficult to derive from conventional multiple logistic regression without additional definition and analysis. We conducted ML analysis to predict patients with severe CDI by utilizing all the variables investigated and demonstrated the top variables selected by the algorithms. Of these, serum albumin, maximum body temperature, and eosinophils were consistent with the predictors identified in previous studies (Kulaylat et al., 2018; McDonald et al., 2018), oxygen saturation, GCS, platelet count, and respiratory rate were the same as those included in the SOFA or qSOFA score. Thus, these components of the SOFA score contributed to the early prediction of severe CDI. In addition, the SOFA score showed a relatively high value in the F1 score, a more informative metric for evaluating predictive models on an imbalanced dataset of the outcome of interest (Saito and Rehmsmeier, 2015), and a fair AUROC value for predicting severe CDI (Hosmer et al., 2013). Therefore, in our data, the SOFA score was as good as the ML models in predicting patient prognosis.

Although we included a large number of CDI cases using an electronic data extraction program, our results are limited by the retrospective and single-country nature of the study. Thus, hidden bias and residual confounders might have influenced the generalization of the results, and PCR ribotyping of non-stored *C. difficile* strains could not be performed. Incomplete sampling may have underestimated the impact of ribotypes on the outcomes of patients with CDI. Furthermore, the hospitals participating in the data ranged from secondary to tertiary care centers, and patient populations could be inherently different. However, we tried to analyze risk factors for severe CDI by minimizing selection bias and multicollinearity using a PS-matched study and ML techniques.

Since the clinical course and outcomes of CDI are highly variable, from uncomplicated diarrhea to surgical intervention or death, predictive indicators of severe CDI are required at diagnosis. The SOFA score is a well-validated model in many clinical settings, based on standardized and early obtainable parameters. Even after controlling for other variables using PS-matching analysis, we found that the SOFA score was a clinical predictor of severe CDI. We also demonstrated that the use of quinolones in the hospital setting could be associated with the bacterial ribotype in patients with CDI because of antibiotic resistance.

## Data Availability Statement

The raw data supporting the conclusions of this article will be made available by the authors, without undue reservation.

## Ethics Statement

The studies involving human participants were reviewed and approved by the Institutional Review Board at Severance Hospital, affiliated with the Yonsei University Health System (3-2021-0508), approved this study. Written informed consent for participation was not required for this study in accordance with the national legislation and the institutional requirements.

## Author Contributions

MHC analyzed the data. MHC and HK wrote the manuscript. DK, SHJ, HML, and HK collected the samples and clinical data. DK, SHJ, and HML critically read the manuscript. All authors contributed to the article and approved the submitted version.

## Conflict of Interest

The authors declare that the research was conducted in the absence of any commercial or financial relationships that could be construed as a potential conflict of interest.

## Publisher’s Note

All claims expressed in this article are solely those of the authors and do not necessarily represent those of their affiliated organizations, or those of the publisher, the editors and the reviewers. Any product that may be evaluated in this article, or claim that may be made by its manufacturer, is not guaranteed or endorsed by the publisher.

## Supplementary Material

Supplementary Figure 1 Described progression-free survival curves for three types of SOFA SCORES [including changes in SOFA score **(A)**, quick SOFA **(B)**, and SOFA **(C)**].

Supplementary Figure 2 Critical variables with important plot for predicting severe CDI. Feature importance including feature weight, mean gain, and coverage of eXtreme gradient boost.

Supplementary Figure 3 Annual incidence of fluoroquinolone prescriptions per 1,000 inpatient-day.
