# Supplementary figures and images for: Risk Factors of Severe Clostridioides difficile Infection; Sequential Organ Failure Assessment Score, Antibiotics, and Ribotypes
Source: Front Microbiol. 2022 May 12;13:900681. doi: 10.3389/fmicb.2022.900681 (PMC9133954; doi:10.3389/fmicb.2022.900681)

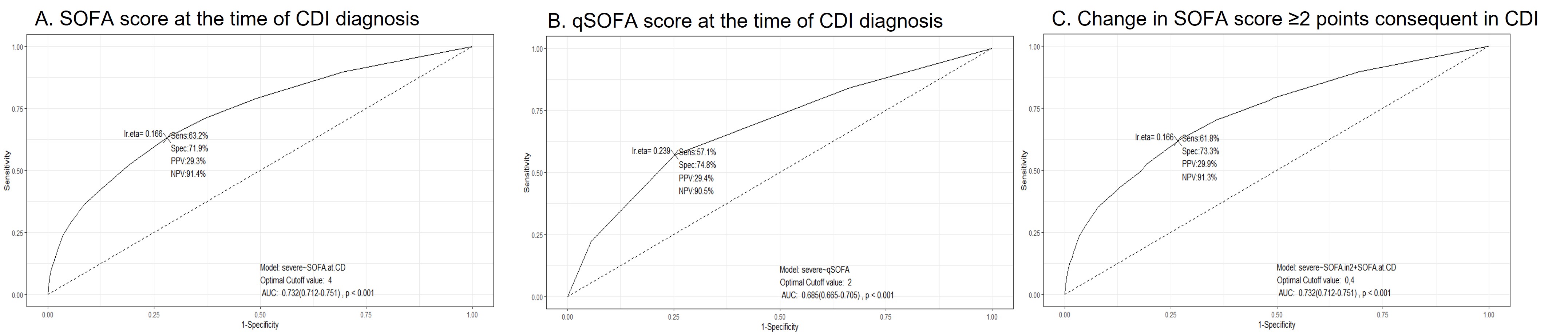

Supplement: Supplementary Figure 1 — Described progression-free survival curves for three types of SOFA SCORES [including changes in SOFA score (A), quick SOFA (B), and SOFA (C)]. [file Image_1.JPEG]

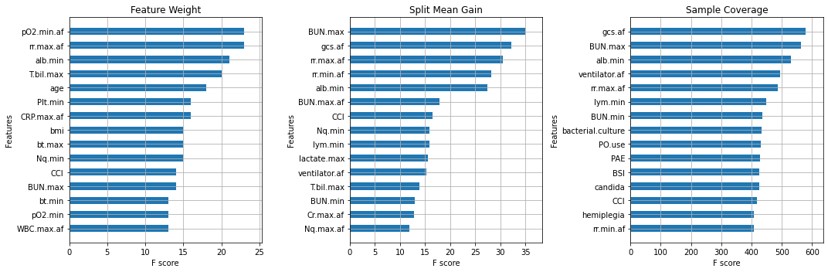

Supplement: Supplementary Figure 2 — Critical variables with important plot for predicting severe CDI. Feature importance including feature weight, mean gain, and coverage of eXtreme gradient boost. [file Image_2.JPEG]

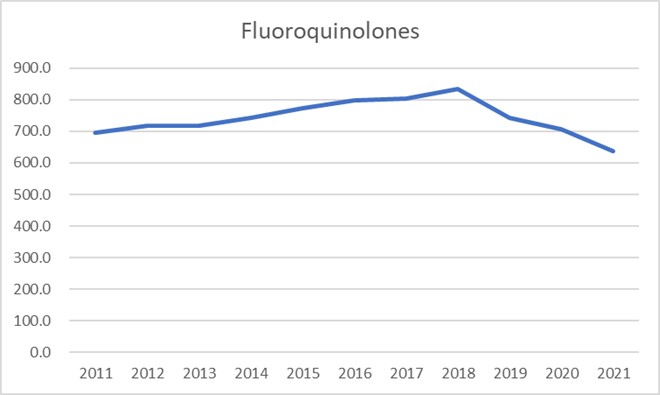

Supplement: Supplementary Figure 3 — Annual incidence of fluoroquinolone prescriptions per 1,000 inpatient-day. [file Image_3.JPEG]
