# Supplementary material for: Risk Factors of Severe Clostridioides difficile Infection; Sequential Organ Failure Assessment Score, Antibiotics, and Ribotypes
Source: Front Microbiol. 2022 May 12;13:900681. doi: 10.3389/fmicb.2022.900681 (PMC9133954; doi:10.3389/fmicb.2022.900681)
Supplement: Supplementary file 4 [file Table_1.docx]

Supplementary Table 1. Performance metrics for the SOFA scores and machine learning classifiers

| CDI severity prediction models | AUROC | Accuracy | F1 score |
| --- | --- | --- | --- |
| SOFA score | 0.732 (0.712-0.751) | 0.706 | 0.400 |
| Quick SOFA | 0.685 (0.665-0.705) | 0.721 | 0.388 |
| Increased in SOFA score ≥2 points | 0.732 (0.712-0.751) | 0.715 | 0.403 |
| **Machine learning models** |  |  |  |
| К-nearest neighbor | 0.645 (0.608-0.682) | 0.834 | 0.169 |
| Light gradient boosting | 0.801 (0.770-0.832) | 0.859 | 0.341 |
| Decision tree | 0.712 (0.679-0.747) | 0.833 | 0.331 |
| Random forest | 0.800 (0.769-0.830) | 0.857 | 0.331 |
| eXtreme gradient boosting | 0.806 (0.776-0.834) | 0.854 | 0.386 |
| Artificial neural network | 0.780 (0.759-0.801) | 0.852 | 0.378 |
| Support vector machine | 0.748 (0.715-0.781) | 0.848 | 0.110 |

Abbreviations: AUROC, area under the receiver operating characteristic curve; SOFA, sequential organ failure assessment
